# The RFK catalytic cycle of the pathogen *Streptococcus pneumoniae* shows species-specific features in prokaryotic FMN synthesis

**DOI:** 10.1080/14756366.2018.1461857

**Published:** 2018-04-25

**Authors:** María Sebastián, Adrián Velázquez-Campoy, Milagros Medina

**Affiliations:** a Facultad de Ciencias, Departamento de Bioquímica y Biología Molecular y Celular, and Instituto de Biocomputación y Física de Sistemas Complejos (BIFI) (GBsC-CSIC and BIFI-CSIC Joint Units), Universidad de Zaragoza, Zaragoza, Spain;; b Fundación ARAID, Diputación General de Aragón, Zaragoza, Spain;; c Aragon Institute for Health Research (IIS Aragon), Zaragoza, Spain

**Keywords:** FAD synthetase, riboflavin kinase, stopped-flow, isothermal titration calorimetry, *Streptococcus pneumoniae*

## Abstract

Emergence of multidrug-resistant bacteria forces us to explore new therapeutic strategies, and proteins involved in key metabolic pathways are promising anti-bacterial targets. Bifunctional flavin-adenine dinucleotide (FAD) synthetases (FADS) are prokaryotic enzymes that synthesise the flavin mononucleotide (FMN) and FAD cofactors. The FADS from the human pathogen *Streptococcus pneumoniae* (*Spn*FADS)–causative agent of pneumonia in humans − shows relevant catalytic dissimilarities compared to other FADSs. Here, by integrating thermodynamic and kinetic data, we present a global description of the riboflavin kinase activity of *Spn*FADS, as well as of the inhibition mechanisms regulating this activity. Our data shed light on biophysical determinants that modulate species-specific conformational changes leading to catalytically competent conformations, as well as binding rates and affinities of substrates versus products. This knowledge paves the way for the development of tools − that taking advantage of the regulatory dissimilarities during FMN biosynthesis in different species − might be used in the discovery of specific anti-pneumococcal drugs.

## Introduction

*Streptococcus pneumoniae* is the causative agent of human pneumonia disease^1^, meningitis and bacteremia in children and adults. It is estimated that 1.6 millions of people, including 1 million of children under age five die every year of pneumonia disease^2^^,^^3^. The irruption during the last decades of multi-drug resistant pneumococci has revealed the need of finding new drugs, as well as novel drug targets. The bifunctional flavin-adenine dinucleotide (FAD) synthetase (FADS) from *S. pneumoniae* (*Spn*FADS) arises as a potential drug target^4^^,^^5^, since it synthesises the essential cofactors flavin mononucleotide (FMN) and FAD^6^, involved in a plethora of vital processes as part of flavoproteins and flavoenzymes^7–9^. As other bacterial FADSs, *Spn*FADS produces FMN and FAD through two sequential activities. First, a riboflavin kinase activity (RFK) at its C-terminus module phosphorylates riboflavin (RF) to FMN, and then the adenosine 5′-triphosphate (ATP):FMN:adenylyltransferase (FMNAT) activity of the enzyme N-terminus module transforms FMN into FAD^10–12^. Two characteristics stand out among the suitable properties of FADSs as drug targets. First, bacterial FADSs differ from the human proteins that synthesise FMN and particularly FAD. Thus, the eukaryotic FMNAT activity is catalysed by an enzyme with a completely different protein folding and chemistry relative to the N-terminus of bacterial FADS^13–16^. Regarding FMN production, the monofunctional *Homo sapiens* RFK has been hardly characterised so far. Overall, it is structurally homologous to the RFK module of bacterial enzymes, but only structures containing bound ligands are available^17^^,^^18^. Nonetheless, structural data predict differences in conformational changes to achieve the catalytic complex^19^^,^^20^, and the scarce biochemical information suggests differences in redox environmental requirements for maximal activity^11^^,^^12^. Second, the members of the prokaryotic FADSs family studied up to now differ catalytically among them, which might facilitate the design of new species-specific medicines. Structurally, when comparing *Spn*FADS with the member of the family so far best characterised—which is that from the organism *Corynebacterium ammoniagenes* (*Ca*FADS)—it presents a very similar structure, with little differences in the position of some key loops. However, these two proteins only share the 26% of sequence homology^6^. Despite the overall structural similitude among prokaryotic FADSs^6^^,^^12^^,^^18^, *Spn*FADS shows three main distinctive functional behaviors; (i) it mainly stabilises monomers—which are the functional form^6^—or traces of dimers, during catalysis; (ii) its FMNAT activity requires reduced FMN as a substrate; and (iii) its RFK activity is not regulated by the RF substrate^6^.

Here, we focus on the *Spn*FADS RFK activity, using pre-steady and steady-state biophysical techniques to describe this activity and the inhibition mechanism employed by this enzyme to regulate FMN synthesis. We take a close look at the thermodynamic and kinetic basis that determine the ligand binding order, the co-operativity between ligands and the inhibitory mechanism performed by the reaction products. Furthermore, we compare our results with those obtained for the FADS from *C. ammoniagenes* to identify key regulatory differences between both proteins.

## Methods

### Cloning, expression, and purification of SpnFADS

*Spn*FADS was cloned, overexpressed, and purified as previously described^6^. In short, *Escherichia coli* Bl21 Star™ (DE3) cells were transformed with a pET-15b vector that contains the DNA sequence encoding *Spn*FADS. Transformed cells were grown and protein expression induced through isopropyl β-D-1-thiogalactopyranoside (IPTG) addition. Cells were harvested and broken by sonication. The supernatant was loaded into a His-Trap affinity column and the protein eluted applying a 10–500 mM imidazole gradient. The His_6_-Tag was removed and then the protein was loaded into HisTrap HP and GSTrap 4B connected columns. The unbound fraction was further purified by size exclusion chromatography. The protein purity was tested and pure protein aliquots were conserved at −80 °C.

### Steady-state RFK activity

The *Spn*FADS RFK activity was measured at 25 °C in 500 µL of 20 mM 1,4-piperazinediethanesulfonic acid (PIPES) and 0.8 mM MgCl_2_, pH 7.0. Reaction samples contained different concentrations of RF (0.5–30 µM) and ATP (10–500 µM), as previously described^21^^,^^22^. The inhibitory effect of the products of the reaction was analysed as previously described^23^. In short, the *Spn*FADS RFK activity was determined at increasing concentrations of FMN, varying the ATP concentration and keeping the RF constant (when studying the inhibitory effect of FMN), and at increasing concentrations of adenosine 5′-diphosphate (ADP), varying the RF concentration and keeping the ATP fixed (when studying the inhibitory effect of ADP). The flavin composition of the supernatant was determined using an Alliance high performance liquid chromatography (HPLC) system (Waters, Milford, MA, USA) equipped with a 2707 autosampler and a HSST3 column (4.6 × 50 mm, 3.5 mm; Waters) preceded by a precolumn (4.6 × 20 mm, 3.5 mm; Waters) as previously described^21^^,^^22^. The FMN concentration was quantified using its standard curve. All the experiments were carried out in triplicate.

Michaelis–Menten (*K*_m_) and catalytic rate (*k*_cat_) constants were obtained by fitting the obtained data to the Michaelis equation^24^. The inhibitory mechanism performed by the products of the RFK reaction − FMN and ADP − was analysed by evaluating their effects on the *K*_m_ and *k*_cat_ values, obtained by the individual fitting of data sets to the Michaelis–Menten model. Additionally, the data sets were globally fit utilising the Lineweaver–Burk equations for mixed inhibition^25^ (Equation (1)).

(1) $$\frac{1}{V_{0}}=\frac{\left(1+\frac{\left[I\right]}{K_{i}}\right)\cdot K_{\text{m}}}{V_{max}}\frac{1}{\left[S\right]}+\frac{\left(1+\frac{\left[I\right]}{{K^{\prime }}_{i}}\right)}{V_{max}}$$

where, [S] and [I] are the concentration of substrates and product inhibitor, respectively. *K_i_*and *K’_i_* are the product inhibition constants^25^. Experiments were performed in triplicate. The estimated errors in *k*_cat_, *K*_m,_ and *K_i_* were within ±15% of their values.

### Pre-steady-state kinetics

Kinetic experiments in the pre-steady state were registered as previously described^23^, using stopped-flow spectroscopy on an Applied Photophysics SX17. MV spectrophotometer, using the Xscan software (Applied Photophysics Ltd., Leatherhead, UK). Fast kinetic measurements were carried out as previously described^23^, at 25 °C in PIPES 20 mM pH 7.0, 0.8 mM MgCl_2_. About 0.2 µM *Spn*FADS was mixed with reaction samples that contained increasing concentrations of the flavin ligands (FLV, herein indicating RF or FMN), in the presence and in the absence of ADP or ATP (herein referred as ANP). Controls were measured in the same buffer but without MgCl_2_. All concentrations indicated here are the final ones in the reaction cell. The kinetic traces were registered until obtaining three reproducible traces.

Kinetic traces were fit to exponential equations (Equation (2)), where each exponential term describes a different process. A linear correction term was added (Equation (3)) when a specific process was not finished within the measuring timeframe.

(2) $$y=\sum {A_{i}e}^{-k_{obs,i}.t}$$

(3) $$y=\sum {A_{i}e}^{-k_{obs,i}.t}+bt$$

where, *A_i_* and *k*_obs__,_*_i_* are the amplitude and the observed kinetic constant for each process (*i*) that contributes to the overall time-dependent fluorescence change.

The processes whose *k*_obs_ showed a linear dependency on the flavin concentration were fit to a one-step model that describes the kinetic equilibrium for the formation and the dissociation of enzyme-flavin complex (Equation (4))

(4) $$k_{obs}=k_{on}\left[\mathrm{FLV}\right]+k_{off}$$

where, *k*_on_ and *k*_off_ are the complex formation and dissociation kinetic constants, respectively.

Experiments were performed in triplicate. The flavin photobleaching was analysed as previously described^23^ (not shown).

### Isothermal titration calorimetry

We performed isothermal titration calorimetry (ITC) assays to elucidate both the ligand binding order and the thermodynamic inhibition produced by the binding of the FLV and ANP ligands. Titrations were performed on an AutoITC200 (MicroCal, Malvern, UK) thermostated at 20 °C, as previously described^23^. Approximately 25 µM *Spn*FADS contained in a 200 µL-cell was titrated with solutions of 180 µM RF, 250 µM FMN, or 350 µM ATP or ADP. Additionally, titrations with ANP ligands into mixtures that contain the protein pre-bound to FLV ligands were performed, as well as titrations with FLV ligands into pre-bound *Spn*FADS:ANP complexes. The titrations were conducted through 19 stepwise injections of 2 µL of the titrating ligand to the calorimetric cell, as previously described^21^^,^^26^. Both the ligands and the protein were dissolved in 20 mM PIPES, pH 7.0, either in presence of 0.8 mM MgCl_2_ or in absence of this cation, and degassed before the titration. The enthalpy change (Δ*H*), the association constant (*K_a_*), and the binding stoichiometry (N) were obtained through non-linear least squares regression of the data using a homemade fitting routine corresponding to a model for one or two independent binding sites, implemented in Origin 7.0 (OriginLab, Northampton, MA, USA), as previously described^21^^,^^26^. The Gibbs free energy (Δ*G*), the entropic contribution (−TΔ*S*), and the dissociation constant (*K_d_*) were obtained using well-known thermodynamic equations.

Cooperativity constants (α) between ANP and FLV ligands were obtained as previously described^23^^,^^27^^,^^28^. Particular titrations with ANP ligands into mixtures of the protein and 100 µM FLV were fit to a homemade fitting routine corresponding to a model that considers the influence of the FLV ligand in the protein binding affinity for the ANP ligand^27^^,^^28^.

Experiments were performed in triplicate. The errors considered in the measured parameters (±15% in *K*_d_ and *K*_a_ values, ±0.3 kcal mol^−1^ in Δ*G*, Δ*H,* and −TΔ*S* and ±20% in α) were assumed to be larger than the standard deviation between replicates and the numerical error after fitting analysis.

## Results and discussion

### The products of the reaction inhibit the RFK activity of SpnFADS

**Figure 1.**
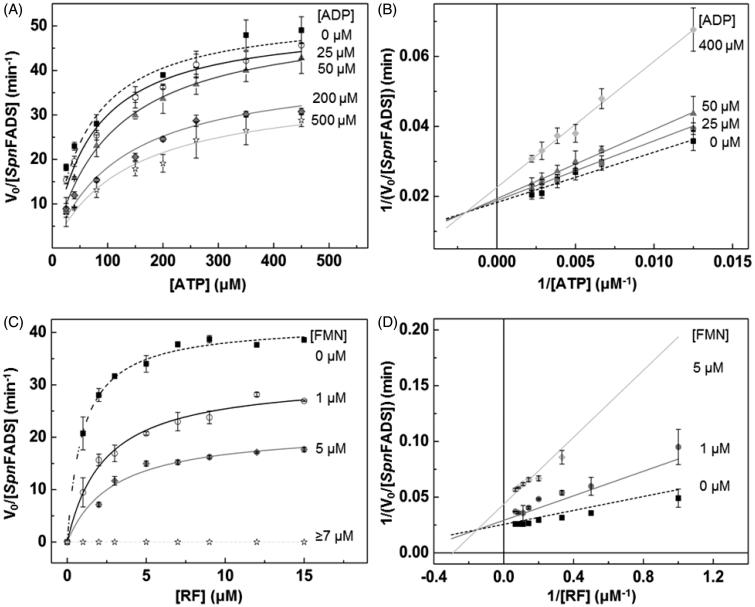
Inhibition of the *Spn*FADS RFK reaction by FMN and ADP products. Michaelis–Menten plots as a function of (A) ATP and (C) RF substrates at different concentrations of the ADP and FMN products. Lineaweaver–Burk representation at different (B) ADP and (D) FMN concentrations with global fitting to the equation for mixed inhibition.

Feedback inhibition is a frequent strategy to regulate enzymes involved in key metabolic pathways^29^^,^^30^. Some bacteria regulate FMN synthesis through the inhibition of the RFK activity of their bifunctional FADSs by the reaction products–FMN and ADP–and/or the RF substrate^19^^,^^23^, although the inhibition level triggered differs between organisms^6^^,^^23^. Here, our Michaelis–Menten plots of the *Spn*FADS RFK activity, for both reaction substrates–RF and ATP–at increasing concentrations of the FMN and ADP products, reveal that *k*_cat_ decreases while *K*_m_^ATP^ and *K*_m_^FMN^ increase (Figure 1). These data point out to mixed or uncompetitive inhibition mechanisms. Therefore, to discern the inhibition mechanism, we carried out Lineweaver–Burk representations (Figure 1(B,D)), which reveal that both products act as mixed inhibitors (Figure 1). Thus, FMN and ADP bind to the free enzyme as well as to the *Spn*FADS:ATP and *Spn*FADS:RF complexes, respectively. However, their affinities for the free protein and for the complex considerably differ (Table 1), being *K_i_*^ADP^ and *K_i_*^FMN^ 6.4 and 5.5 times smaller than *K´_i_*^ADP^ and *K´_i_*^FMN^, respectively. That indicates that both reaction products bind preferentially to the free enzyme. Also, the considerably smaller inhibition constants for FMN compared to ADP (*K_i_*^ADP^/*K_i_*^FMN^ = 100) reveals the flavin product as a much more potent inhibitor (Table 1).

**Table 1.** Steady-state kinetic parameters for the *Spn*FADS RFK activity as calculated by global fitting to the Lineweaver–Burk equation.

*K_i_*^ADP^ (μM)	*K_i_*′^ADP^ (μM)	*k*_cat_ (min^−1^)	*K*_m_^ATP^ (μM)	*K_i_*^FMN^ (μM)	*K_i_′*^FMN^(μM)	*K*_m_^RF^ (μM)
130 ± 16	844 ± 97	55 ± 2	75 ± 7	1.3 ± 0.4	7.1 ± 1.3	1.2 ± 0.3

Equivalent data obtained for the so far best characterised FADS–that belonging to *C. ammoniagenes* (*Ca*FADS)–show that ADP and FMN are, respectively, competitive and uncompetitive inhibitors of its RFK activity^23^. The FMN product appears as a considerable more potent inhibitor of the RFK activity of *Ca*FADS than of *Spn*FADS, as the *K*_m_^RF^/*K_i_*^FMN^ values of 7.1 and 0.8 for *Ca*FADS and *Spn*FADS, respectively, show. This, together with the fact that RF strongly inhibits the *Ca*FADS RFK activity but not the *Spn*FADS one^6^, points to species-specific inhibition and activity modulation mechanisms. Physiologically, other preferred regulatory strategies, such as the use of reduced FMN during the FMNAT activity, and the different oligomeric assemblies established along both activities of these enzymes^6^^,^^31^^,^^32^, might be also behind the distinct requirements for the RFK activity regulation.

### The binding of the substrates of the RFK reaction is the fastest and most favored process for SpnFADS

Then we used stopped-flow spectrophotometry^23^^,^^33^ to kinetically identify individual steps during the *Spn*FADS RFK reaction. Here, we took advantage of two aspects; (i) RF and FMN have the same fluorescence spectra and yields^34^ and (ii) under oxidising conditions flavins only bind and get transformed at the RFK module of *Spn*FADS^6^. Consequently, although RF would be transformed into FMN, we would only observe fluorescence changes derived from flavin binding, flavin dissociation, and/or conformational changes at the RFK module.

**Figure 2.**
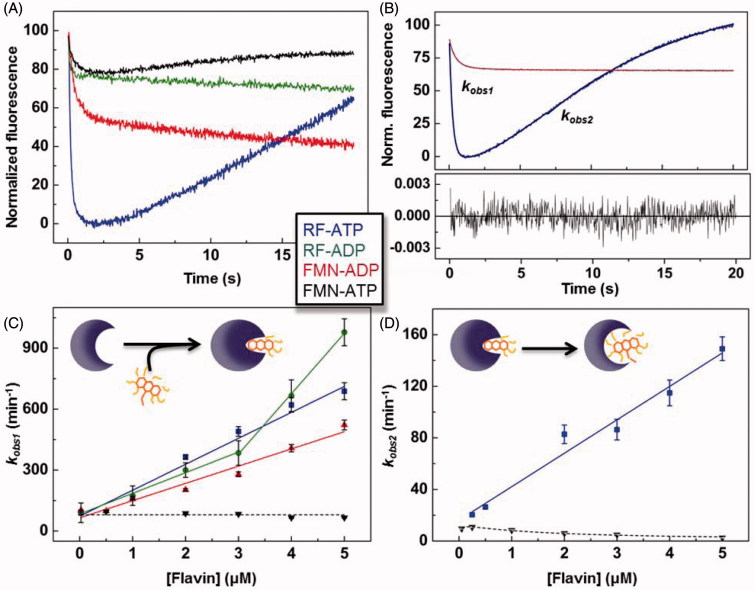
Pre-steady-state stopped-flow kinetics for RF and FMN binding to *Spn*FADS in the presence of adenine nucleotides. (A) Kinetic traces for the flavin fluorescence evolution upon mixing the protein with ANP-FLV combinations. (B) Example of fittings of kinetic traces. Evolution of (C) *k*_obs1_ and (D) *k*_obs2_ on FLV concentration, with schemes representing the corresponding processes in the insets.

When mixing *Spn*FADS with RF or FMN (herein FLV) ligands, we only observed flavin photobleaching, similarly to that reported for *Ca*FADS^23^. This suggests that *Spn*FADS is not able either to bind oxidised flavins or to internalise their isoalloxazine ring. On the contrary, fast and intense exponential flavin fluorescence variations were observed when mixing *Spn*FADS simultaneously with ANP (ATP or ADP) and FLV ligands. All samples showed fast initial fluorescence decays, but mixtures containing ATP also presented subsequent fluorescence increases (Figure 2(A,B)). As recently reported for *Ca*FADS^23^, we relate the initial fluorescence decay to FLV binding and internalisation through a conformational change of the loop-L4c, that closes the flavin binding site when ANP is previously bound (Supplementary Figure SP1)^20^. The succeeding fluorescence raise can be similarly related to an ATP-induced conformational change that re-opens the flavin binding site, making the isoalloxazine ring accessible again to the solvent^23^ (Figure 2(C,D), schemes). *k*_obs1_ and *k*_obs2_ represent, respectively, the observed rates for these processes. The linear dependence of *k*_obs1_ on FLV concentrations (Figure 2(C)) allows us calculating flavin association and dissociation rate constants (*k*_on_ and *k*_off_, respectively) and the derived dissociation constants (*K_d_*). The binding of the RFK substrates–RF and ATP–is the fastest process (Table 2), and also shows the largest fluorescence decay amplitude (Figure 2(A)). Hence, contrary to that reported for *Ca*FADS^23^, the *Spn*FADS RFK site preferably binds the RF and ATP substrates over other combinations of ligands, being the binding of the FMN product in presence of the ATP substrate the least favored combination. In addition, *k*_obs2_ values show that the conformational rearrangement for flavin release is considerably faster when RF is the flavin initially bound (Figure 2(D)). Moreover, *k*_obs2_ shows a biphasic behavior negatively affected by the FMN concentration, indicating that accumulation of FMN hinders its release. Noticeable, *k*_obs1_ and *k*_obs2_ for processes that involve RF and ATP are in the *k*_cat_ range, revealing that the reaction steps represented by these parameters are relevant for catalysis. Nevertheless, as the *k*_obs1_/*k*_obs2_ ratio indicates, the binding and the internalisation of the reaction substrates is 4 times faster than the subsequent conformational change that releases flavins to the solvent, being this last process the *Spn*FADS RFK reaction bottleneck.

**Table 2.** Pre-steady-state kinetic parameters for flavins binding and dissociation to *Spn*FADS in the presence of adenine nucleotides.

Ligands combination	*k*_on_ (min^−1^·μM^−1^)	*k*_o__f__f_ (min^−1^)	*K_d_* (μM)	Δ*G* (kcal·mol^−1^)
FMN-ADP	85 ± 7	65 ± 20	0.76 ± 0.24	−8.3 ± 2.5
FMN-ATP	^a^	^a^		
RF-ADP	102 ± 6^b^	84 ± 11^b^	0.83 ± 0.12	−8.3 ± 1.4
RF-ATP	128 ± 14	73 ± 16	0.57 ± 0.14	−8.5 ± 2.0

a *k*_obs1_ values close to 0 prevented determination of *k*_on_ and *k*_off_.

b Values obtained when [RF] ≤ 3 μM. When [RF] ≥ 3 μM, *k*_on_ ∼ 297 ± 9 and *k*_o__f__f_ could not been determined.

**Table 3.** Cooperativity coefficients for the binding of the different combinations of FLV and ANP ligands to *Spn*FADS in Mg^2+^ absence.

Ligands	α	*N*	Δ*h* (kcal mol^−1^)
FMN-ADP	0.35 ± 0.06	0.40 ± 0.03	−58 ± 3
FMN-ATP	1.3 ± 0.2	1.1 ± 0.1	−0.20 ± 0.05
RF-ADP	0.7 ± 0.1	0.9 ± 0.1	2.1 ± 0.1
RF-ATP	1.4 ± 0.1	1.9 ± 0.04	−58 ± 1

α is the cooperativity coefficient, *N* the fraction of total protein able to bind the titrating ligand, and Δ*h* the enthalpy change associated to each process.

Considering our results in the context of those reported for the RFK cycle of *Ca*FADS, some key facts are worthy to be highlighted. Both proteins require ANP nucleotides to bind/internalise flavin ligands, and they show the same overall individual processes along the reaction. Additionally, both FADSs are able to bind all the possible combinations of ANP-FLV ligands, although differences in the relative binding rates and magnitude of the associated spectroscopic changes must be behind the lower inhibition levels shown by *Spn*FADS. Thus, while binding of the substrates of the RFK reaction—that is RF and ATP—is the fastest and most favored process for *Spn*FADS (Table 2), it is the slowest one for *Ca*FADS^23^. This fact together with a 10-times higher *K_i_*^ADP^, and a 9-times higher *K*_m_^RF^/*K_i_*^FMN^ ratio for *Ca*FADS than for *Spn*FADS, explains the larger inhibition by the reaction products observed for *Ca*FADS (Table 1)^23^. Comparison of the *k*_obs2_ behavior for both enzymes (Figure 2(D))^23^—*k*_obs2_ linearly depends on the RF concentration for *Spn*FADS while it shows a biphasic behavior for *Ca*FADS—shows that during the *Ca*FADS RFK reaction, high RF concentrations inhibit the ATP-induced conformational change for flavin release, while *Spn*FADS does not present such inhibition. This might be a determinant of the strong inhibition by RF that *Ca*FADS suffers^21^^,^^23^, which is absent in *Spn*FADS.

### Thermodynamics explain the modest inhibition of the SpnFADS RFK activity

**Figure 3.**
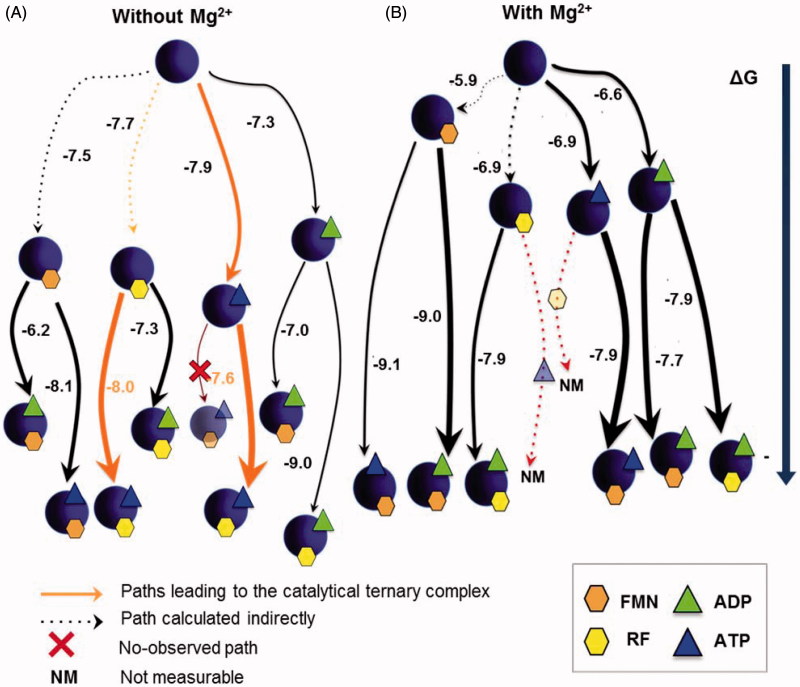
Gibbs free energy diagrams for the interaction of *Spn*FADS with its ligands. Titrations performed (A) without MgCl_2_, and (B) with 0.8 mM MgCl_2_. Numbers indicate the *ΔG* value in kcal mol^−1^. The arrows thickness is proportional to the protein fraction able to bind the ligand. NM denotes paths where the reaction heat would mask the binding enthalpy.

We next used ITC to determine whether the identified kinetic processes were relevant to reach the thermodynamic equilibrium. We titrated with the substrates and the products of the RFK reaction, both free *Spn*FADS and its binary mixtures with either ANP or FLV ligands. Since Mg^2+^ is necessary for the RFK reaction to occur, we performed all titrations with and without this cation, to determine its role on ligands binding. However, titrations that involve both reaction substrates only were executed without Mg^2+^, since the reaction heat would mask the binding enthalpy (Figure 3(A)).

Figure 3 illustrates all the possible binding pathways occurring in the interaction landscape of the *Spn*FADS RFK module with the substrates and products. As reported for *Ca*FADS^23^, Mg^2+^ is key in the formation of *Spn*FADS:ANP:FLV complexes (Figure 3 and Supplementary Table SP1): (i) by increasing the protein fraction prone to interact with a specific ligand, and consequently, the probability of the pathway, and (ii) by favoring the formation of ternary complexes from the binary ones (compare arrows thickness and *ΔG* values in Figure 3(A,B)). The stabilisation of these ternary complexes by Mg^2+^ is mainly a consequence of the less unfavorable binding entropy (Supplementary Table SP1), which suggests different conformations in ternary complexes with and without MgCl_2_. It is worthy to highlight that although FLV binding to the free protein is not directly observed by ITC or stopped-flow spectrophotometry, flavins—particularly when Mg^2+^ is present—highly modify the protein affinity for ANP ligands (Figure 3(B), Supplementary Table SP1). This cooperative effect—also observed with *Ca*FADS^23^—evinces that RF and FMN act as slow-binding ligands^35^. However, although their binding is too slow to be measured within the experimental time, it can be indirectly estimated^27^^,^^28^^,^^36^.

Titrations without MgCl_2_ (Figure 3(A)) allow establishing an interaction diagram that includes “pseudo-reactive” pathways with the reaction substrates (*Spn*FADS:ATP:RF). Comparison of this diagram with that obtained for *Ca*FADS^23^ reveals important differences, which might contribute to the regulatory dissimilarities displayed between both proteins. Thus, (i) two alternative pathways lead to the *Spn*FADS:ATP:RF complexes, while *Ca*FADS presents only one pathway^23^ and (ii) *Spn*FADS:ATP:RF complexes are almost the most stable ones—as their lowest position in the *ΔG* diagram indicates—and also the most probable—thickest orange arrows—. This situation is completely different for *Ca*FADS, where all the other non-productive *Ca*FADS:ANP:FLV complexes are favored against *Ca*FADS:ATP:RF^23^.

We can extract two main conclusions from these results. First, both proteins achieve the catalytic FADS:RF:ATP complex through different mechanisms; *Spn*FADS seems to follow a random sequential binding of the RFK substrates, while *Ca*FADS requires its concerted fit^23^. Second, as the RFK reaction progresses and the products accumulate, pathways different from the reactive one are favored in the case of *Ca*FADS, which reduces the efficiency of the overall catalysis^23^. For its part, the pathway that leads to *Spn*FADS:ATP:RF is the most favored, at least until reaching a high [products]/[substrates] ratio.

### Conformational differences explain the dissimilar regulation of prokaryotic RFK cycles

**Figure 4.**
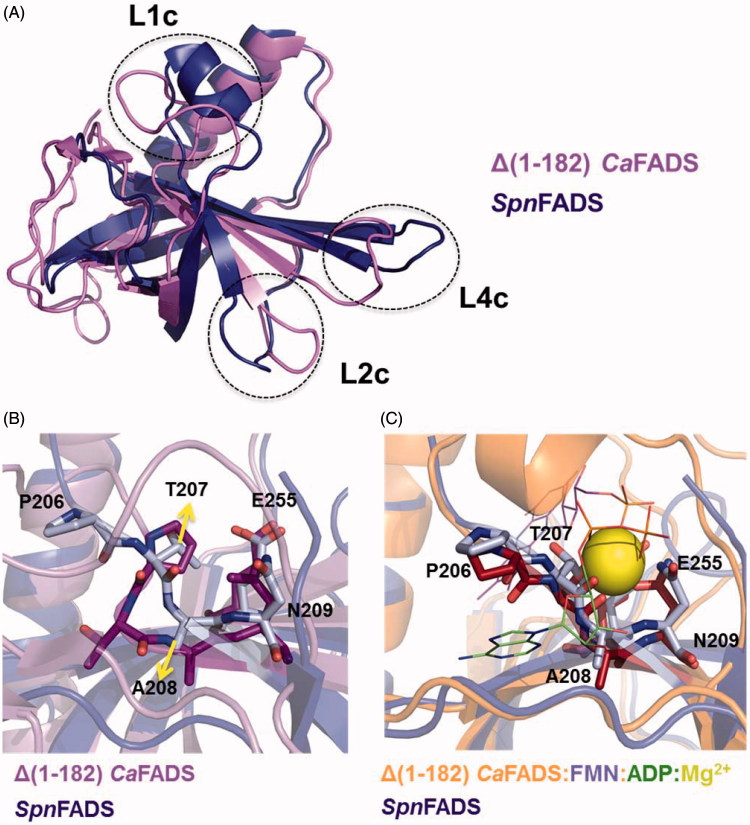
The C-terminal modules of *Ca*FADS and *Spn*FADS. (A) Superposition of the RFK modules of *Spn*FADS (PDB 3OP1) and *Ca*FADS (PDB 2X0K). Zoom into the conformation of the PTAN motif of *Spn*FADS and the corresponding residues in (B) *Ca*FADS and (C) *Ca*FADS:ADP:FMN (PDB 5A89).

Structurally, the different mechanisms by which RF and ATP get allocated in the RFK active site might be related to the simpler conformational changes required by *Spn*FADS. Figure 4 shows the relative conformation of the catalytic PTAN motif in *Spn*FADS regarding *Ca*FADS (Figure 4(B)) and *Ca*FADS:FMN:ADP structures (Figure 4(C)). Remarkably, the conformation of this motif in *Spn*FADS resembles that of *Ca*FADS in a ternary complex with the products of the RFK reaction. So, the ligand-induced conformational change of this motif, which is crucial for the *Ca*FADS RFK cycle^20^, is not necessary for *Spn*FADS. This allows flexibility in the allocation order of substrates in the *Spn*FADS RFK active site.

Figure 3 also reveals highly different *ΔG* values for titrations of either free *Spn*FADS or of its binary complexes with the same ligand, as well as that flavins are able to bind the enzyme in the presence of ANP ligands but not in its absence. These effects can be explained considering binding cooperative effects between ANP and FLV ligands (Table 3), and slow binding of flavins to free *Spn*FADS. Therefore, we carried out a co-operativity study that again came out with fundamental differences regarding heterotropic cooperativity in the *Ca*FADS RFK module. Our data show that for *Spn*FADS the RF and ATP substrates always show positive cooperativity (Table 3). On the contrary, in the case of *Ca*FADS low RF concentrations facilitate ATP binding, while increasing RF amounts hinder it^23^. This different cooperative behavior of the substrates explains the inhibition by the RF substrate that *Ca*FADS shows but not *Spn*FADS. Structurally, such effect might be also related to the different conformation of the PTAN motif. In *Ca*FADS, the occupation of the ANP binding site by RF —when it is in excess— might prevent the ligand-induced conformational change of this motif, which is necessary for ATP binding (Figure 4)^23^. This structural rearrangement is not necessary for *Spn*FADS (Figure 4(B)), and therefore, the excess of RF does not hinder ATP binding.

Collectively, our results shed light on the kinetic and thermodynamic basis behind the substrates and products inhibition regulatory differences in the RFK cycles of *Spn*FADS and *Ca*FADS. These essential enzymes differ in the substrates binding order, as well as in the inhibitory potency of the products and their action mechanism. The lesser inhibition level presented by *Spn*FADS—regarding *Ca*FADS—is because of the binding of the reaction substrates is the fastest and thermodynamically most favored process, regardless of products accumulation. The structural origin of such divergences is the dissimilar conformation that the key PTAN motif shows in both FADSs, which points to species-specific conformational changes during the RFK activity. This fact, together with other differential regulatory strategies, such as the use of reduced substrates, provide us with a broader frame in which we could work in the development of new anti-microbials specific for *S. pneumoniae.* That way, it might be feasible to envisage drugs binding to a particular *Spn*FADS conformation, being unable to interact–due to the structural constraints and functional differences–with other FADSs.

## Conclusions

In conclusion, here we present a complete description of the RFK catalytic cycle of *Spn*FADS, integrating thermodynamic and kinetic data, both in the pre-steady and in the steady-state, obtained using different biophysical and biochemical tools. We consider our research highly relevant in a double way. On one hand, combining physico-chemical tools commonly used in the biophysical characterisation of proteins, we have designed a strategy to go beyond the information directly extracted from this kind of techniques to offer a model that contributes to explain the dynamics during the *Spn*FADS RFK catalytic cycle. On the other hand, the results derived from our study are also relevant for the scientific community. Thus, we have found important differences between the RFK catalytic cycles of two structurally similar essential proteins, i.e. *Ca*FADS and *Spn*FADS, shedding light on the thermodynamic and kinetic determinants that led to these inhibitory differences. Taking into account the pathogenic character of *S. pneumoniae,* the essentiality of its FADS and the last tendencies in discovering species-specific drugs, which reduce the resistances emergence, the knowledge here presented might facilitate the development of drugs able to bind a specific conformation of *Spn*FADS, having no effect on other members of the FADS family.

## Supplementary Material

IENZ_1461857_Supplementary_Material.pdf
