# Supplementary material for: The RFK catalytic cycle of the pathogen Streptococcus pneumoniae shows species-specific features in prokaryotic FMN synthesis
Source: J Enzyme Inhib Med Chem. 2018 Apr 25;33(1):842–9. doi: 10.1080/14756366.2018.1461857 (PMC6010069; doi:10.1080/14756366.2018.1461857)
Supplement: IENZ_1461857_Supplementary_Material.pdf [file IENZ_A_1461857_SM9619.pdf]

Supplemental Information

The RFK catalytic cycle of the pathogen *Streptococcus pneumoniae* shows species-specific features in prokaryotic FMN synthesis

María Sebastián[⁺], *Adrián Velázquez-Campoy*[⁺,‽,▲], *Milagros Medina*[⁺,*].

[⁺] Departamento de Bioquímica y Biología Molecular y Celular, Facultad de Ciencias, and Instituto de Biocomputación y Física de Sistemas Complejos (BIFI) (GBsC-CSIC and BIFI-CSIC Joint Units), Universidad de Zaragoza, Spain

[‽] Fundación ARAID, Diputación General de Aragón, Spain

[▲] Aragon Institute for Health Research (IIS Aragon), Zaragoza, 50009, Spain

*Correspondence to: Milagros Medina. Departamento de Bioquímica y Biología Molecular y Celular. Facultad de Ciencias. Pedro Cerbuna 12. Universidad de Zaragoza. 50009-Zaragoza. Spain. Fax: +34 976 762123; Phone: +34 976 762476 e-mail: mmedina@unizar.es

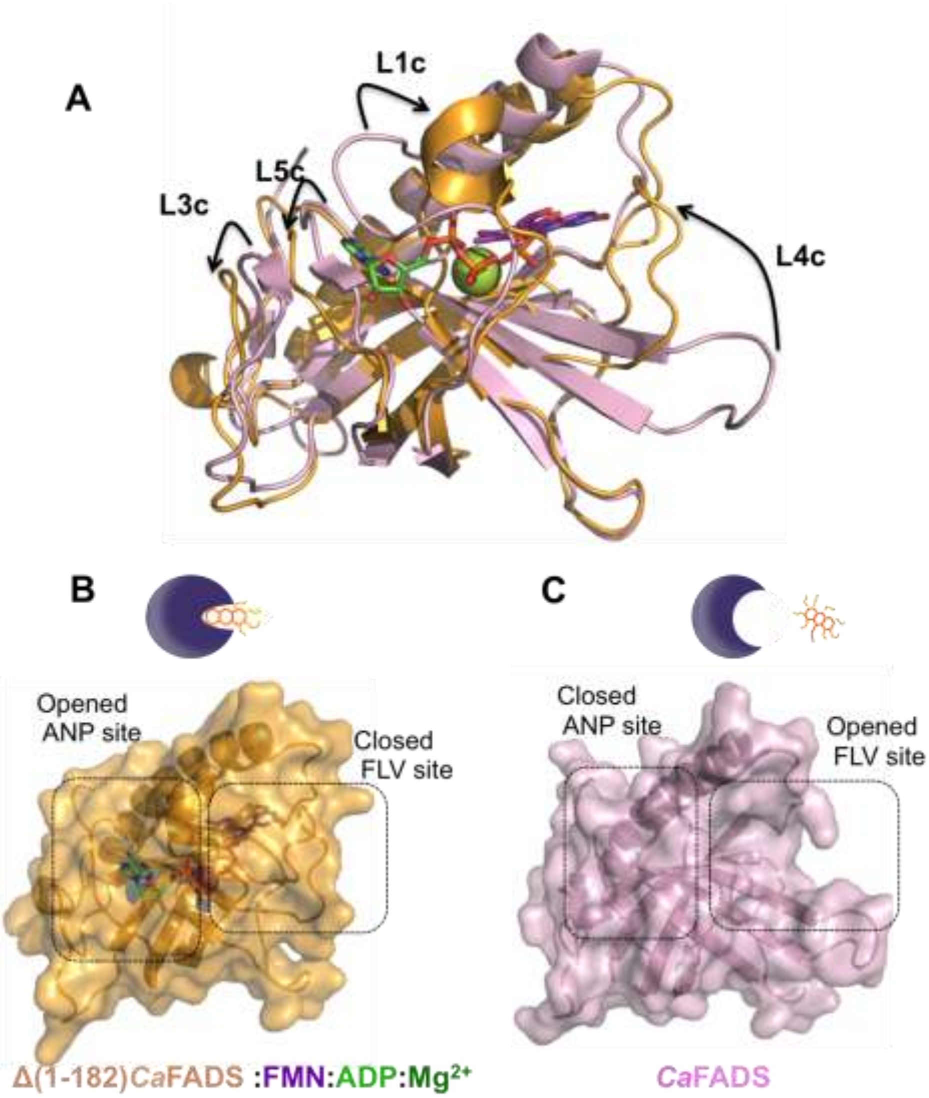

Figure SP1. Crystallographic structures of the *Ca*FADS RFK module, free (pink, PDB 2x0k) and in complex with the products FMN and ADP:Mg^{2+} (orange, PDB 5a89). A) Superposition of both structures. B) Detail of the adenine nucleotide and the flavin binding sites (ANP and FLV, respectively), in the ternary complex. C) Detail of the adenine nucleotide and the flavin binding sites in the free RFK module. ADP and FMN ligands are shown as sticks in CPK color with carbons in green and purple respectively, and the Mg^{2+} cation is shown as a green sphere. Purple circles schematize the fluorescence of the flavin ring when free and when quenched upon binding to the protein.

Table SP1. Thermodynamic interaction parameters for the combination of *Spn*FADS with different ligands as obtained through ITC. Column named initial mixture indicates the starting composition of the calorimetric cell. Titrations were carried out at 20ºC in 20 mM PIPES, pH 7.0 (upper table) and in 20 mM PIPES, pH 7.0, 0.8 mM $MgCl_2$ (lower table). Errors in ΔG, ΔH and $-T\Delta S$ were estimated in ± 0.3 kcal/mol and in ±15% in K_d, taken in general larger than the standard deviation between three replicates and the numerical error after fitting analysis.

0.0 mM $MgCl_2$					
initial mixture	ligand	K_d (μM)	ΔG (kcal/mol)	ΔH (kcal/mol)	$-T\Delta S$ (kcal/mol)
*Spn*FADS	ATP	1.7	-7.9	-19	11
*Spn*FADS	ADP	4.1	-7.3	-13	5.5
*Spn*FADS	RF	n.d.[a]	n.d.[a]	n.d.[a]	n.d.[a]
*Spn*FADS	FMN	n.d.[a]	n.d.[a]	n.d.[a]	n.d.[a]
*Spn*FADS:FMN	ADP	28	-6.2	-26	19
*Spn*FADS:RF	ATP	1.2	-8.1	-12	3.5
*Spn*FADS:FMN	ADP	28	-6.2	-26	19
*Spn*FADS:FMN	ATP	1.2	-8.1	-16	7.5
*Spn*FADS:ATP	FMN	n.d.[a]	n.d.[a]	n.d.[a]	n.d.[a]
*Spn*FADS:ATP	RF	2.5	-7.6	-1.9	-5.7
*Spn*FADS:ADP	FMN	7.9	-7.0	-22	15
*Spn*FADS:ADP	RF	0.3	-9.0	-17	8.2

0.8 mM $MgCl_2$					
initial mixture	ligand	K_d (μM)	ΔG (kcal/mol)	ΔH (kcal/mol)	$-T\Delta S$ (kcal/mol)
*Spn*FADS	ATP	8.1	-6.9	-12	4.9
*Spn*FADS	ADP	14	-6.6	-7.0	0.4
*Spn*FADS	RF	n.d.[a]	n.d.[a]	n.d.[a]	n.d.[a]
*Spn*FADS	FMN	n.d.[a]	n.d.[a]	n.d.[a]	n.d.[a]
*Spn*FADS:RF	ATP [b]				
*Spn*FADS:RF	ADP	1.7	-7.9	-17	9.2
*Spn*FADS:FMN	ADP	0.24	-9.0	-7.8	-1.2
*Spn*FADS:FMN	ATP	0.23	-9.1	-17	8.1
*Spn*FADS:ATP	FMN	1.6	-7.9	-6.0	-1.9
*Spn*FADS:ATP	RF [b]	n.m. [b]	n.m. [b]	n.m. [b]	n.m. [b]
*Spn*FADS:ADP	FMN	2.3	-7.7	-8.1	0.4
*Spn*FADS:ADP	RF	1.6	-7.9	-6.9	-1.0

[a] n.d. Not detected. Not heat of interaction was detected for this titration. [b] n.m. Not measured. This combination of ligands in the presence of Mg^{2+} leads to the catalytic reaction, preventing determination of binding heats
